# Exercise for Health and Disease: Time to Move Ahead

**DOI:** 10.1155/2017/1460262

**Published:** 2017-08-21

**Authors:** Paulo Gentil, Fabrício Boscolo Del Vecchio, James Steele

**Affiliations:** ^1^Faculdade de Educação Física e Dança, Universidade Federal de Goiás, Goiânia, GO, Brazil; ^2^Escola Superior de Educação Física, Universidade Federal de Pelotas, Pelotas, RS, Brazil; ^3^School of Sport, Health, and Social Sciences, Southampton Solent University, Southampton, UK

We are pleased to present this special issue. As we noted in our call for papers for this special issue,

the volume of exercise science research increases every year; however, it is disappointing to note that exercise prescription has continued to follow the same guidelines for many decades. Have we not uncovered any new findings that would make exercise prescription more efficient and overcome many of the purported barriers to participation? Is there no evidence to help health professionals to adequately choose and design exercise program for specific outcomes?

Physical inactivity is considered one of the most important public health problems of the 21st century [1]. Indeed, mortality due to physical inactivity is as high as tobacco smoking [2]. The failure to reach minimal amounts of physical activity decreases life expectancy by 3–5 years [3] and increases the risk of cancer, heart disease, stroke, and diabetes up to 30% [3, 4]. Despite its effect in prevention, exercise has also an important role in treating diseases, being considered as a polypill, due its wide positive effects [5]. Regular practice of exercise contributes to body mass control, improvement in muscle health, and reduction on body fat percentage. Nevertheless, the prevalence of sedentarism is alarming [6] and the percentage of overweight and obese people is increasing [7].

It is important to recognize that the positive effects of exercise are null if people do not engage with it and if the programs engaged with do not produce improvements in the desired outcomes [8, 9]. In this sense, we expect that this special issue can improve our knowledge concerning different aspects of the relationship between exercise, health, and disease. Here we present seven articles which have considered varied exercise approaches across a range of populations, both healthy and diseased, and in varied contexts.

Contributions from A. Wittke et al., W. D. N. Santos et al., and J. Steele et al. have considered applications of resistance training exercise in both healthy (middle-aged males and elderly adults) and diseased populations (breast cancer survivors). They have provided insight into the applications of resistance training (application of progressive high effort), its effects in combination with supplementation (protein), and both the positive outcomes and risk of adverse effects in a clinical population (breast cancer survivors).

Work from C. Ranucci et al., T. Dalager et al., and L. Fox et al. have also offered insights into “real world” multidisciplinary approaches to exercise. C. Ranucci et al. report the positive effects of a family-based multidisciplinary approach to improving health status, nutrition habits, and physical performance in overweight and obese children or adolescents. T. Dalager et al. showed the implementation of “Intelligent Physical Exercise Training” compared with moderate intensity physical activity on a workplace setting upon musculoskeletal health. Further, L. Fox et al. provide important “real world evidence” on quantitative and qualitative data feedback from men with prostate cancer who had undergone a structured exercise intervention.

Lastly, S. C. E. Schmidt et al. report on the results of an important 18-year longitudinal study examining the effects of physical activity types, fitness, and health in adults. They report key findings regarding the role of type of physical activity upon fitness and health, as well as the impact of confounding sociodemographic factors.

We hope that the contributions from authors in this special issue serve to aid in enhancing specific exercise prescription in a range of populations and that they also stimulate further interest and work in advancing our understanding of exercise in both health and disease.

*Paulo Gentil* *Fabrício Boscolo Del Vecchio* *James Steele*
